# ‘The Drug Survey App’: a protocol for developing and validating an interactive population survey tool for drug use among Aboriginal and Torres Strait Islander Australians

**DOI:** 10.1186/s13722-022-00298-2

**Published:** 2022-03-14

**Authors:** James H. Conigrave, Scott Wilson, Katherine M. Conigrave, Tanya Chikritzhs, Noel Hayman, Angela Dawson, Robert Ali, Jimmy Perry, Michelle S. Fitts, Louisa Degenhardt, Michael Doyle, Sonya Egert, Tim Slade, Nadine Ezard, Monika Dzidowska, K. S. Kylie Lee

**Affiliations:** 1 The University of Sydney, Faculty of Medicine and Health, Central Clinical School, NHMRC Centre of Research Excellence in Indigenous Health and Alcohol, Camperdown, Australia; 2 The Edith Collins Centre (Translational Research in Alcohol Drugs and Toxicology), Sydney Local Health District, Camperdown, Australia; 3 Aboriginal Drug and Alcohol Council (South Australia) Aboriginal Corporation, Underdale, Australia; 4 Royal Prince Alfred Hospital, Drug Health Services, Camperdown, Australia; 5 National Drug Research Institute, Faculty of Health Sciences, Curtin University, Perth, Australia; 6 Southern Queensland Centre of Excellence in Aboriginal and Torres Strait, Islander Primary Health Care, Inala, Australia; 7 School of Medicine, University of Queensland, Herston, Australia; 8 School of Medicine, Griffith University, Gold Coast, Australia; 9 Faculty of Health, University of Technology Sydney, Sydney, Australia; 10 Faculty of Health and Medical Sciences, University of Adelaide, North Adelaide, Australia; 11 Charles Darwin University, Menzies School of Health Research, Alice Springs, Australia; 12 Western Sydney University, Institute for Culture and Society, Parramatta, NSW Australia; 13 University of New South Wales, National Drug and Alcohol Research Centre, Kensington, Australia; 14 Faculty of Medicine and Health, Matilda Centre for Research in Mental Health and Substance Use, The University of Sydney, Camperdown, Australia; 15 Alcohol and Drug Service, St Vincent’s Hospital, Darlinghurst, Australia; 16 Burnet Institute, Melbourne, Australia; 17 Centre for Alcohol Policy Research, La Trobe University, Melbourne, Australia

**Keywords:** Substance use, Illicit drug use, Brief intervention, Aboriginal and Torres Strait Islander, Tablet survey

## Abstract

**Background:**

Disadvantage and transgenerational trauma contribute to Aboriginal and Torres Strait Islander (Indigenous) Australians being more likely to experience adverse health consequences from alcohol and other drug use than non-Indigenous peoples. Addressing these health inequities requires local monitoring of alcohol and other drug use. While culturally appropriate methods for measuring drinking patterns among Indigenous Australians have been established, no similar methods are available for measuring other drug use patterns (amount and frequency of consumption). This paper describes a protocol for creating and validating a tablet-based survey for alcohol and other drugs (“The Drug Survey App”).

**Methods:**

The Drug Survey App will be co-designed with stakeholders including Indigenous Australian health professionals, addiction specialists, community leaders, and researchers. The App will allow participants to describe their drug use flexibly with an interactive, visual interface. The validity of estimated consumption patterns, and risk assessments will be tested against those made in clinical interviews conducted by Indigenous Australian health professionals. We will then trial the App as a population survey tool by using the App to determine the prevalence of substance use in two Indigenous communities.

**Discussion:**

The App could empower Indigenous Australian communities to conduct independent research that informs local prevention and treatment efforts.

## Introduction

Alcohol and other drug use is a major cause of death and illness internationally [1]. Due to legacies from British colonisation, Aboriginal and Torres Strait Islander peoples (‘Indigenous Australians’) experience broad socioeconomic disadvantage, and often transgenerational trauma and discrimination [2]. This disadvantage has far-reaching consequences [3]. Indigenous Australian communities are actively working to reduce harms from alcohol and other drug use [4]. While the majority of Indigenous Australians are not at risk from alcohol and other drug use [5, 6], they are more likely to have adverse health outcomes from substance use than other groups [7]. But the prevalence of drug use varies greatly within and between communities [5, 8, 9], and over time [10]. Regular, accurate estimates of local drug use patterns are needed to plan tailored interventions and enable equitable resource allocation. While culturally appropriate methods to measure alcohol consumption among Indigenous Australians have been established [11, 12], validated and acceptable tools which measure illicit or extra-medical drug use patterns (amount used, frequency of use, and dependence symptoms [13]) are needed.

Collecting reliable self-report data on alcohol and other drug use is complex in any population [14, 15]. Drug use can vary in the frequency (regularity) and the intensity of use (the amount consumed per occasion). The frequency of drug use can be determined from participant recall. The intensity of use can be harder to estimate for drugs because a given drug can vary in concentration or can be consumed in a variety of ways (e.g., methamphetamines can be smoked, or injected) which influence the total bio-availability, the speed of metabolism, experiences, and consequences [16–18].

Multiple quantity-frequency measures have been validated for alcohol [19], but most available screening tools do not collect data on the intensity at which illicit or extra-medical drugs are consumed. Instead, they collect data on the frequency of drug use and the frequency of symptoms of dependence and other harms (e.g. Indigenous Risk Impact Screen [20], and ASSIST (Alcohol, Smoking and Substance Involvement Screening Test) [21]). While difficult to collect, data on the amount of illicit and extra-medical substances consumed is important. Harms increase when larger amounts of a given drug are taken more frequently (e.g. overdose [22], dependence [16], mental health disorders [23–26], and risk-taking behaviours including dangerous driving [27] and unsafe sex [28]). Accurately determining drug concentration from participant self-report may not be possible, but estimating intensity using average drug concentrations may still be suitable for population surveys of drug use.

Perceptions of privacy and confidentiality are important considerations for Indigenous Australian survey respondents, particularly when personal data is collected [15]. The Australian government has a history of committing human rights violations against Indigenous Australians, including the forced removal of Indigenous children from their families [29], and racially targeted restrictions of Indigenous self-determination [30]. Some respondents might be fearful that admissions of illicit or extra-medical drug use to an interviewer would be passed on to authorities with serious consequences such as child removal [15, 31]. To ensure accurate reporting, survey respondents must be confident that their responses are confidential and private [14, 15, 31].

Most existing surveys of alcohol and other drug use are designed for non-Indigenous populations. Differences in language, lifestyle and cultural contexts might make these tools less acceptable or inaccurate when used with Indigenous respondents [31]. For example, some Indigenous participants may feel more comfortable describing their quantity of drug use as their share of a group’s use, rather than their individual use [15]. Drug names and terminology vary greatly between Indigenous Australian communities and geographical locations [32]. Surveys that refer to drugs using their chemical names rather than brand, common or street names may be misunderstood in some Indigenous Australian contexts.

Recently, interactive survey applications (‘apps’) have received attention for their ability to address many barriers which prevent some Indigenous Australians from accurately describing their alcohol consumption [15, 31]. For example, the ‘Grog Survey App’ (developed by some members of the current research team) [12] uses tablet technology to read out questions in plain English and in Pitjantjatjara (an Indigenous Australian language spoken in parts of South Australia, Western Australia, and the Northern Territory). Survey respondents can describe the type of alcohol consumed, what it was mixed with (e.g. soft drink), and what container it was consumed from (e.g. cans, wine glasses, or slushie cups) [12, 33]. Respondents can describe their consumption as a proportion of what their whole group drank. The App automatically converts reported alcohol consumption to standard drinks (each of 10 g alcohol [34]). The Grog Survey App has been found to be a valid and acceptable tool to help Indigenous Australians describe how much they drink [11, 12, 35].

But no similar tools exist for Indigenous Australians to describe illicit or extra-medical drug use. Such tools are needed as there is ongoing concern about the increasing use of drugs such as methamphetamines in some Indigenous Australian communities [36, 37]. In this paper, we present a protocol for co-designing and validating a tablet-based tool (‘The Drug Survey App’), to measure alcohol and other drug use patterns. We also describe the design of an electronic brief intervention which will be included in the App but which will be evaluated as part of a separate project.

## Method

### Project overview

**Fig. 1:**
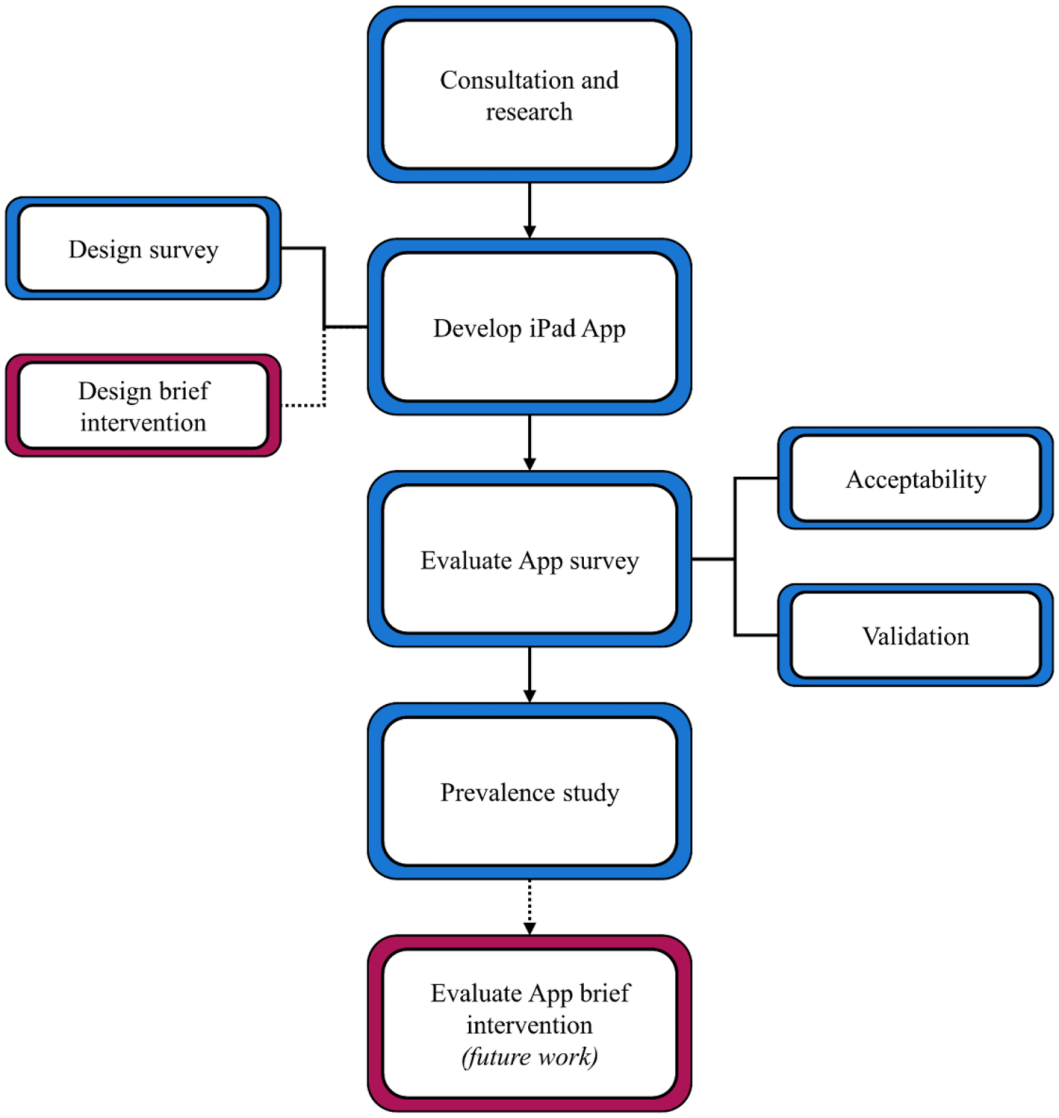
Study flow diagram. The current study includes four distinct phases: consultation, development, evaluation, and the prevalence study. The brief intervention will be evaluated as part of a future study

This study builds upon previous work on the Grog Survey App [12]. The study will include four phases: consultation, development, evaluation, and a prevalence study (Fig. 1). The App will first be co-designed with Indigenous and non-Indigenous Australian researchers and clinicians, Indigenous community members, and a contracted company to write the software. Following the development, a validation study will be conducted where responses to the App are compared to those given in clinical interviews. An evaluation will be conducted to assess the acceptability of the App for Indigenous Australian respondents. The Drug Survey App will then be trialled as a survey tool to obtain prevalence estimates of use in two Indigenous Australian communities (one remote and one urban). We will design a brief intervention to include in the App which will be evaluated as part of a separate project.

### Ethical approval

Ethical approvals to conduct the study have been received from the Aboriginal Health Council of South Australia (#04-20-874); Aboriginal Health and Medical Research Council of New South Wales (#1716-20); and Metro South Health, Queensland Health (#HREC/2021/QMS/73775).

### Indigenous Australian leadership

This project was co-conceived by an Indigenous Australian health professional (SW) and a non-Indigenous researcher (KL). The Drug Survey App will be co-designed with stakeholders including Indigenous Australian health professionals, addiction medicine specialists, community leaders, and researchers (both Indigenous and non-Indigenous Australian). The Indigenous Advisory Group of the Centre of Research Excellence in Indigenous Health and Alcohol will provide guidance for the study.

### App design

The App will be designed as a Progressive Web App (PWA) which allows for development across multiple platforms [38]. The App will be made available on Android tablets, iPads, and desktop computers. The App can collect data without an internet connection. Once an internet connection is resumed, the App will upload responses to a secure server at the University of Sydney. The App will ask questions about participants’ demographics, alcohol and extra-medical drug use patterns (frequency and intensity of use, and symptoms of dependence), harms experienced from alcohol and other drug use (to self or others), treatment access, and how easy and acceptable they found the App to use. The survey structure and App assets will be stored in a content management system (built in ‘Umbraco’ [39]) which will enable researchers to modify survey questions and App content based on research objectives. The content management system will also facilitate downloading data in CSV and JSON format from University servers, and the generation of HTML reports which describe alcohol and other drug use among survey respondents.

### Stakeholder engagement and expert consultation

A mock-up of the survey tool will be developed in Microsoft PowerPoint. Investigators, clinicians, and Indigenous community members will be asked to provide feedback and to suggest improvements (individually or in small groups). This prototype will be updated by the lead investigator (KL) based on feedback and then sent back to stakeholders iteratively until consensus is reached. To accommodate the unpredictability of COVID-19 restrictions in Australia, a series of online forums will be convened with a broader group of stakeholders (n ≈ 30; Australia-wide) including Indigenous Australian health professionals and community members, Indigenous and non-Indigenous Australian researchers and App developers. The forum will facilitate discussion on how best to ask Indigenous Australians about drug use and dependence. All App elements (including question wording) will be reviewed by Indigenous Australian researchers, health professionals and community members to ensure suitability for diverse Indigenous Australian contexts.

### App components

#### Elements

**Fig. 2:**
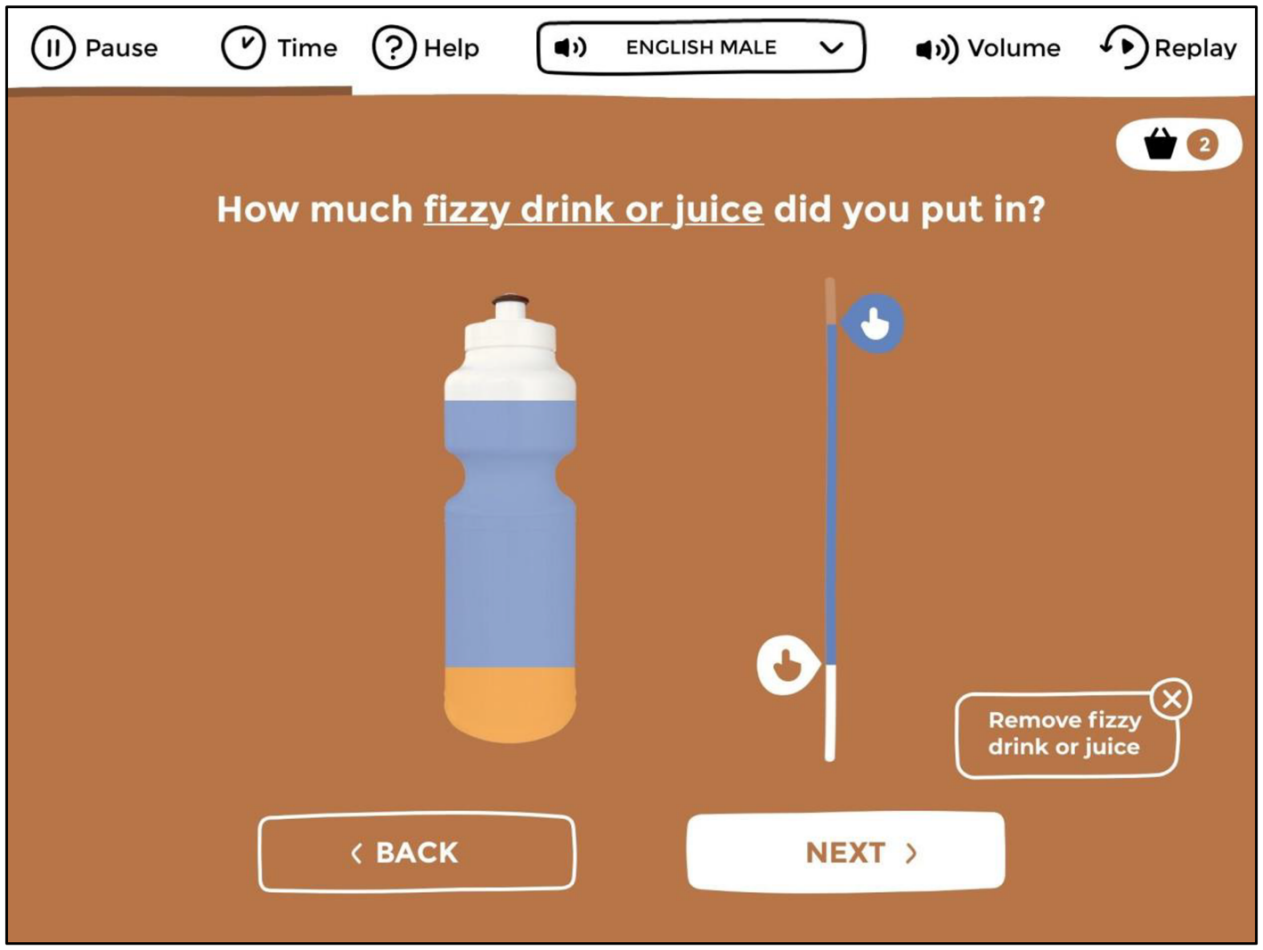
An example of the kind of interactive interfaces that will be used in the Drug Survey App. This image is a screenshot from the Grog Survey App upon which the Drug Survey App builds. Participants can drag sliders to indicate the amounts of whisky and soft drink that were placed inside a water bottle

**Fig. 3:**
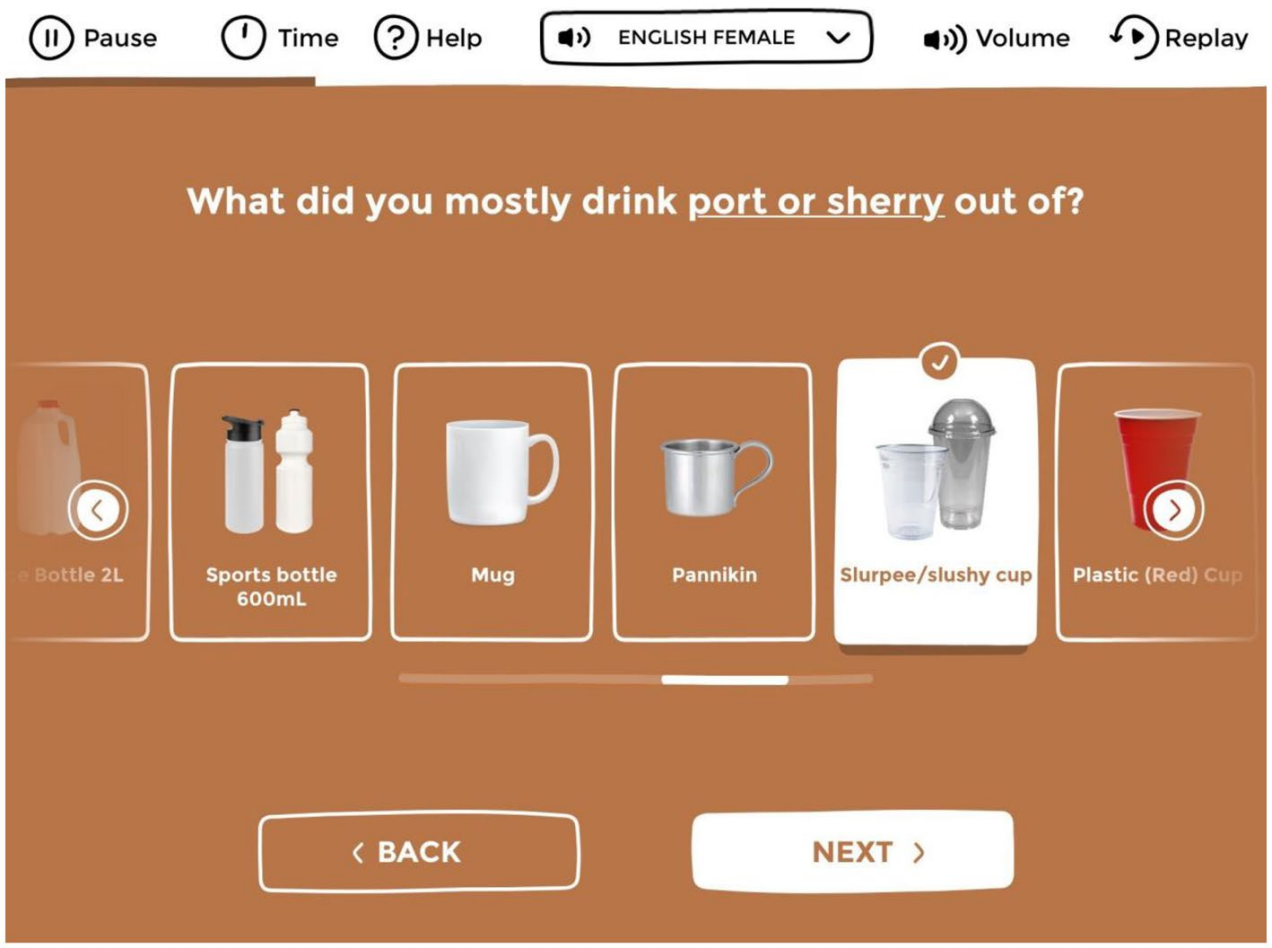
Carousels allow for participants to select from a list of responses which may not all fit on a single screen. To see additional options, participants can either touch the arrow keys, or swipe along the responses on screen

The App will be co-designed to be flexible, such that the questions and content included can change depending on the priorities of the research team, community or health service. User interfaces will be optimised for touch screens. For instance, sliders will be used to report on continuous variables (e.g. age, quantity of a given drug; see Fig. 2), and ‘carousels’ for selecting items from longer lists which would otherwise not fit on smaller screens (see Fig. 3). Iterative user testing will ensure the App’s suitability for diverse Indigenous contexts. All App questions and response categories will be voice-narrated. Survey respondents can select whether they want questions to be read out by a man or woman, and in English or in a local Indigenous language (we will build the functionality for an unlimited number of Indigenous languages to be supported, but at launch only Pitjantjatjara translations will be implemented). Images shown throughout the App will be matched to the gender selected by the participant at the start of a survey (e.g., women will see images of other women).

#### Demographics

Survey respondents will report on their age, gender, community, income, education, pregnancy status, and employment.

#### Illicit and extra-medical drug use

Illicit and extra-medical drug use will be measured using a new instrument that estimates frequency of use, intensity of use (in the last 12 months), and frequency of dependence symptoms (ICD-11) [13]. A set of questions will establish consumption patterns for each substance. Where possible, items will be adapted from existing scales of drug consumption such as the Drug Use Disorders Identification Test (DUDIT) [40], the Fagerström test for Nicotine Dependence [41], and the Alcohol Smoking and Substance Involvement Screening Test (ASSIST) [21]. The final item content will be determined via iterative stakeholder consultation.

#### Included drugs

Target drugs were determined based on the priorities of Indigenous Australian health professionals and community members (including SW, JP, NH). In addition to alcohol (currently featured on the Grog Survey App), drugs selected were tobacco, cannabis, methamphetamines, heroin, opioid-based painkillers, and benzodiazepines. We will also collect data on other drugs (but without custom images or customised user interfaces). Participants can select drugs from an additional list derived from the National Drug Strategy Household Survey 2019 (e.g. cocaine, ecstasy, steroids, kava, inhalants) [6]. Alternatively, participants can type in the name of any drug which is not listed. Participants will only be asked about the frequency at which they used a drug listed under ‘other’.

#### Alcohol consumption

The Drug App will incorporate sections for measuring alcohol consumption from the Grog App which were previously found to be acceptable and valid [11, 12]. These sections include an adapted ‘Alcohol Use Disorders Identification Test for Consumption’ (AUDIT-C) [42] that visualises responses in participants' preferred beverage; and an interactive visual tool which assists participants in reporting how much alcohol they consumed (in standard drinks) on the last two drinking occasions [11, 12, 35].

### Brief intervention

The Drug app will include a brief intervention, adapted and refined from one previously included in the Grog App. On survey completion, each person will receive a ‘tailored’ brief intervention via the Drug App. Feedback will be provided to participants based on their survey responses. This intervention will be informed by the client-centred motivational interviewing framework FLAGS (Feedback, Listen, Advice, Goal setting, Strategies) [43] and the Indigenous Australian Social and Emotional Wellbeing model [44]. The App will reflect user responses back to participants using colloquial language (e.g., “You told us you are using meth daily, and that if you don’t smoke you feel crook”). As participants can have different motivational frameworks, users can select topics that they care about. The App will then provide information to participants on how their drug-use can affect that domain, and what the benefits of stopping or reducing their current level of use might be. For instance, if a participant selects ‘connecting to culture and community’ they will receive information about how abstaining from drug use could help them engage meaningfully with others in their community. While the brief intervention must be developed alongside the survey (as they are included in the same App) a separate study protocol will be devised for testing its effectiveness as part of a future project.

#### Incorporating feedback from the Grog Survey App

We will shape the brief intervention in the new Drug Survey App based on participant experiences of the Grog Survey App [12]. A Worimi (Indigenous Australian) clinician-researcher and an Anaiwan (Indigenous Australian) research assistant will conduct, face-to-face ‘yarning’ interviews [45] with staff (n = 10) and clients (n = 20) in an inpatient alcohol and other drug residential rehabilitation service in New South Wales, Australia. Yarning interviews are semi-structured interviews which encourage story-telling and relationship building between participants and interviewers to assist in collecting qualitative data on a research topic [45]. Participants will be shown the brief intervention in the Grog Survey App and then asked a series of questions about their experiences. Sample questions include “The sections on alcohol and pregnancy: how could they be improved?” and, “Starting with ice, how can we give feedback on ice use in an App like this?”. Interviews will be audio-recorded and professionally transcribed. Key themes will be identified and described using thematic analysis [46, 47].

### Drug Survey App validation

We will test whether the survey items used to measure patterns of drug use are valid (frequency, amount consumed per occasion, and the frequency of dependence symptoms), acceptable, and reliable. We will enrol participants from South Australia and Queensland with a variety of usage patterns (including participants who never use drugs). As the items for measuring alcohol consumption have been previously found to be valid and acceptable for use with Indigenous Australians [11, 12, 35], they will not be re-tested. Convergent validity of the Drug Survey App will be established by correlating patterns of drug use estimated by the App, to estimates made by Indigenous Australian health professionals in clinical interviews using the timeline followback method [19], and to diagnoses of drug use dependence using ICD-11 criteria [13].

Being surveyed about drug-use might affect patterns of future use [48]. There can also be fluctuations in drug-use patterns over time [10]. Accordingly, differences in reported drug use from the App to the clinical interview might be real, rather than artefactual. To manage this confound, in estimating the validity and reliability of the App we will only consider reports of drug-use which were reported in the common reference period for both the App and clinical interview. For example, if a participant takes the App first, then the clinical interviewers will only ask about drug-use prior to them taking the App. This will prevent changes in behaviour which occur between taking the App and interview from affecting our findings.

#### Clinical interview

Indigenous Australian clinicians will receive training from an Addiction Medicine Specialist (KC). Clinicians will estimate the quantity and frequency of other drug use (in the previous two weeks) using timeline followback and harms experienced related to these. These metrics will then be used by the clinician to establish whether participants are at risk from their other drug use, including likely dependence. Participants will be asked to attend a clinical interview within 2–7 days of taking the App. Approximately half of all participants will attend an interview before using the App, and the other half will complete the App before the interview. Any loss to follow-up will be reported.

#### Reliability

Participants will be invited to again complete the Drug Survey App up to one week after first taking the survey. We will determine the correlation of within-person responses. We will also calculate sensitivity and specificity by comparing first and second survey responses.

#### Acceptability

The Drug Survey App will include three questions to gauge participants’ feedback on completing the survey: “How easy was it to use this iPad to answer our questions?”, “Was it okay for us to ask these questions?”, and “Were the questions easy to understand?”. Responses to these three questions will be on a 5-point Likert scale using emojis (frowning face to smiling face). Response frequencies will be used to describe perceived acceptability. To supplement these metrics, research assistants who administer the app will be trained to collect observational field data in relation to perceived acceptability of the survey, time taken to complete the survey, any practical or technical challenges experienced, the useability of the tablet in different settings (e.g. public venues or community events, private homes). This data will be collected both via the App and through semi-structured debriefing sessions with coordinating site research staff at the end of each data collection day.

#### Eligibility, sample size and stratification

Data collection will occur in two Australian states: South Australia and Queensland. These states were chosen due to existing relationships between the research team and the local communities. Indigenous participants, 16 years and older, will be recruited from primary health care services, outpatient drug treatment, harm reduction services (e.g. need syringe services) and drop in centres. Equal numbers of men and women will be sought. Participants will be evenly recruited from a remote and an urban community (remoteness defined by Australian Bureau of Statistics remoteness classifications [49]). Stratified sampling will be used to capture participants with various substance use patterns. In each state, 20 people who do not use illicit or extra-medical drugs, 40 who use heroin or methamphetamines infrequently, and 40 who use these drugs regularly will be recruited. Given high rates of polydrug use, we expect this stratification will also result in adequate numbers of participants who use cannabis, tobacco, and alcohol. The total sample size will be 200 (Table 1). This sample size will allow us to detect spearman correlations as small as *r* = 0.20 with 80% power.^1^ We will recruit participants through connections with local health and community services.

**Table 1:** Sample stratification for the validation study

	Remoteness		Total
	Urban	Regional/remote	
No illicit or extra-medical drug use	20	20	40
Infrequent illicit or extra-medical drug use	40	40	80
Regular illicit or extra-medical drug use	40	40	80
Total	100	100	200

Remoteness will be derived from Australian standard geographic classification [49]

### Prevalence study

Following validation, we will perform a study of the prevalence of substance use in two Indigenous communities to test the feasibility of using the App as a population survey tool. We aim to describe the prevalence of 12-monthly use of each drug in two Indigenous Australian communities (both in South Australia; one urban and one remote). The sample for the prevalence study will be stratified to match local Indigenous Australian demographics (age, gender, socioeconomic status) based on findings from the most recent published Australian Bureau of Statistics census results. We will work with local Indigenous services to identify appropriate sites and engage hard to reach groups [52]. Any challenges in using the app as a population survey tool will be documented and described.

#### Eligibility and sample size

We will sample all Indigenous participants, 16 years and older. The required sample size is dependent on the magnitude of expected prevalences of substance use and desired level of precision (defined as the distance between an estimate and the upper or lower bound of a 95% confidence interval) [6]. As the proposed survey measures use of a range of drugs, we expect a range of prevalence estimates. Alcohol and cannabis may be frequently used in the last 12 months: a recent meta-analysis estimated that 59% of Indigenous Australians had consumed alcohol [5], and an estimated 24.5% of all Indigenous Australians have smoked cannabis in the past 12 months. We expect other drugs to be used relatively infrequently (according to the National Aboriginal and Torres Strait Islander Health Survey [53]: amphetamines, ice or speed 3.4%; extra-medical use of painkillers or analgesics 3.1%; cocaine 2.9%; heroin < 0.4%).

Based on 2016 census data [54], we expect our urban and remote partner communities to have approximately 1800 and 60 eligible community members, respectively. To estimate power, we used equations described by Arya and colleagues which include a Finite Population Adjustment (FPC) [55]. This adjustment accounts for the added precision of sampling closer to the actual population size [55]. We used a 95% confidence interval in our power calculations. For rare prevalence, accurate estimates can be obtained when precision (d) is two times as small as an estimate (p) [55]. For the urban population, we calculated that if we sampled 800 participants (44.4% of that population), we could estimate prevalence as small as 1.06% with a high degree of precision (p = 0.011; d = 0.005). This sample would enable us to estimate the prevalence for all target drugs except heroin which we expect to be very rare. Given the small number of participants in the remote site, we identified that we need to recruit all eligible community members. We believe that this is feasible, as some research team members reside, work, or have previously conducted research with that community [33].

### Recruitment strategy

We will ask participating services to describe their clients’ characteristics (number, age, gender, and typical severities of substance use). We will use this information to generate a table of potential participants which contains the site they attend, and their characteristics. We will determine the numbers participants needed of each age and gender to make our sample match the Indigenous Australian demographics found at the most recent Australian census. Potential participants will then be selected using stratified random sampling. This sampling frame will be used to generate stratified targets for each sampling site which will be given to research assistants. This strategy has previously been reported as effective for obtaining samples of Indigenous Australians with similar demographics to those expected based on Australian census results [52].

## Discussion

We described a protocol for developing and validating a novel tablet-based population survey instrument for estimating alcohol and other drug use prevalence and patterns of use among Indigenous Australians. If found to be valid and acceptable, this new tool will help communities and researchers measure the prevalence of substance use, dependence, and harms. This App has the potential to empower communities to objectively assess drug use in their own communities. These assessments will help communities to formulate plans to support at-risk community members, and may help them attract funding from external bodies.

Indigenous communities often rely on external research teams to monitor local alcohol and other drug use which requires time, specialised training, and extensive resources. For example, to conduct a ‘simple’ prevalence study, communities would need to locate a valid survey tool, and hire a team of research assistants to complete a wide variety of tasks—interviewing, coding, data entry and data analysis. While collaborations with external research teams *can* be positive, they can also be time consuming, and there is potential for communities to lose control over research questions and the way findings are analysed and disseminated [56].

The Drug App automates many tasks required to conduct prevalence studies. Project leads can set targets for the number of participants to recruit by age and gender. Research assistants can then approach participants and provide them with an iPad loaded with the survey for them to complete. The survey can then be self-administered as participants receive audio instructions in plain English, or a local Indigenous language. Once data are synchronised, communities can then receive a description of their study findings in an infographic with plain English summaries. Accordingly, communities who use the Drug Survey App will have the opportunity to conduct independent research and monitor their progress in reducing alcohol and other drug use and associated harms.

The App might reveal that small numbers of community members are engaging in alcohol and other drug use. This evidence could help challenge stereotypes and help celebrate local efforts to drink safely, and not to use illicit or extra-medical drugs. This community-driven research could also inform local prevention and treatment efforts. Health service administrators could use the App to monitor local alcohol and drug use. Local monitoring could be useful in evaluating the effectiveness of alcohol and drug related initiatives, or responding to changes in usage over time. If conditions are challenging to address locally, then reports produced using the Drug App could be used to attract funding and external support.

In addition to community empowerment, the Drug Survey App might be a useful tool for researchers and policy makers. National surveys of Indigenous alcohol use have been criticised for a lack of cultural appropriateness, or their inability to garner accurate estimates of consumption [31]. This has led to under-reporting of consumption [57, 58] and in turn, fewer resources for prevention and treatment efforts [31, 58]. The App will be co-designed with Indigenous Australian health professionals to ensure that questions suit a variety of Indigenous contexts. Accordingly, the Drug Survey App may be more appropriate for use with Indigenous Australians than surveys with no (or minimal) cultural adaptations. If the Drug Survey App is adopted broadly, then the consistency of survey items and delivery of the App will enable comparisons across regions.

While the Drug Survey App is being designed for Indigenous Australians, many of the challenges in accurately measuring substance use among Indigenous Australians are also faced by researchers measuring substance use in other populations (e.g., culturally and linguistically diverse peoples, or pregnant women). Should the Drug Survey App be validated for Indigenous Australians, it could be adapted for other populations. Such an application could be a powerful way to survey entire populations with the content adapting to the requirements of each participant.

## Limitations

A major limitation in developing population survey tools for Indigenous Australians is a lack of ‘gold standards’. The development of new tools in the future will provide further points of comparison and help identify best practice for measuring drug use among Indigenous peoples. Our validation study over-samples people who frequently use illicit or extra-medical drugs. This is necessary for statistical power. However, the effectiveness of the App as a screening tool could potentially vary for participants with different usage-patterns. The accuracy of participant responses might also be different for regions with more aggressive or relaxed drug policing (which might vary due to laws, police practises, or community attitudes). Accordingly, it would be useful to replicate our studies in different populations, regions and settings. Also, while a brief intervention is included in the App, it will need to be evaluated to test its effectiveness. In particular it is important that the brief intervention is useful to participants regardless of how regularly they use illicit or extra-medical drugs. We hope that the App will help all people understand how to talk about illicit and extra-medical drug use. By doing so, we hope that participants can support family members or friends who use illicit or extra-medical drugs to get help.

## Conclusion

We have presented a protocol to design and validate a novel tablet-based survey to measure alcohol and other drug use among Indigenous Australians. Findings will be reported back to stakeholders and the App will be collaboratively refined. The completed App will be made available for communities, health services, policy makers and researchers. We hope to improve estimates of alcohol and other drug use among Indigenous Australians, so that more funding and support can be garnered to inform local responses.

## Data Availability

The final App will be freely available for Indigenous communities in Australia. Licensing arrangements for the App to be used by non-Indigenous researchers will be determined on a case-by-case basis by the University of Sydney. The University of Sydney reserves the right to charge a fee to help fund App maintenance and server upkeep.

## Footnotes

1 Calculated with the ‘pwr’ library [50] in R version 4.0.5 (2021–03-31) [51].
